# Pulmonary lymphoma mimicking metastases: a case report

**DOI:** 10.1186/1757-1626-2-7081

**Published:** 2009-04-28

**Authors:** Vijay Hadda, Gopi C Khilnani, Ashu Seith Bhalla, Ruchika Gupta, Siddhartha D Gupta, Ashish Goel

**Affiliations:** 1 Department of MedicineAIIMS, New Delhi 110029India; 2 Department of RadiodiagnosisAIIMS, New Delhi 110029India; 3 Department of PathologyAIIMS, New Delhi 110029India; 4 Geriatrics DivisionAIIMS, New Delhi 110029India

## Abstract

**Introduction:**

Lung mass is a common radiological finding among elderly. Bronchogenic carcinoma with metastases is the commonest cause of lung mass with multiple nodules in same or other lung seen in this age group. However, sometimes other uncommon malignancies with better prognosis can also present similarly. Primary pulmonary lymphoma is one of the rare malignancies, which have similar radiological presentation but different treatment and prognosis.

**Case presentation:**

We present a non-smoking, elderly, diabetic man who presented with nonspecific symptoms of generalized weakness without any symptom pertaining to respiratory system. Examination of chest revealed findings suggestive of right lower lobe mass. On evaluation, he was found to have a well circumscribed lung mass on chest radiograph. Computerized tomography of chest with contrast showed a large mass involving right lung and multiple nodules in both lungs. For diagnosis, biopsy from right lung mass was done under image guidance. Histopathology revealed diffuse large B-cell non-Hodgkins lymphoma. Evaluation for primary malignancy leading to lung metastases was inconclusive. Patient was advised chemotherapy.

**Conclusion:**

Primary pulmonary lymphoma is a rare disease and can present with non specific symptoms. Radiologically, it can easily be confused with commoner malignancies like, bronchogenic carcinoma with or without metastases. Primary pulmonary lymphoma carries different therapeutic and prognostic implications. Therefore, physicians should make every effort to achieve histopathological diagnosis before prognosticating patient presenting with lung mass.

## Introduction

Primary pulmonary lymphoma (PPL) is a rare entity. It constitutes less than 1% of NHL in general and 3%-4% of extra-nodal NHL. Among lung malignancies its contribution is only 0.5%-1% [1]. Clinically, these present with non-specific symptoms. Radiologically, these can present as consolidation, well-defined mass or nodules [2-4]. The most common cause of multiple well-circumscribed lesions in elderly is metastases. The primary malignancy is usually in lung, breast or abdomen. So, PPL can easily be confused radiologically with primary lung carcinoma or metastases when presenting as multiple masses or/and nodules. We came across an elderly man with similar radiological picture who was diagnosed primary pulmonary lymphoma. The rare nature of this disease, its non-specific clinical presentation and close mimicry with lung metastases in elderly patients are discussed.

## Case presentation

A 78-year-old Indian, non-smoker man, presented to out patient department with progressively increasing generalized weakness for two months. He gave history (on leading question) of weight loss (about 2 kg) during this period. There was no history of fever, cough, expectoration, hemoptysis or chest pain. Patient denied any history of joint pain, skin rash, photosensitivity, Raynaud's phenomenon or oral ulcers. There was no history high risk sexual behavior, intravenous drug abuse or blood transfusion. Patient had history of type-2 diabetes mellitus since last 10 years and was taking oral hypoglycemic agents (Metformin SR 850 mg twice daily and Glimiperide 2 mg once a day) with good control of blood sugars. He was also suffering from coronary artery disease for which he was taking his medications (Aspirin 75 mg once a day, Metoprolol SR 25 mg twice daily and Atorvastatin 10 mg once a day at night). There was no history of tuberculosis in the past. Family history was non contributory. Physical examination revealed well built and nourished elderly. He was afebrile with oral temperature of 98.2°F. His pulse was 82/minute and blood pressure was 126/72 mm of Hg. There was no pallor, icterus, peripheral lymphadenopathy, clubbing or bony tenderness. Chest examination revealed reduced expansion in infrascapular region on the right side with dull percussion note and reduced breath sounds in the same region. There was no hepatosplenomegaly. Examination of cardiovascular and nervous system did not reveal any abnormality.

Blood investigations revealed hemoglobin of 13.5 gm/dl, total leukocyte count 5,400/µl with differential counts showing mild eosinophilia (neutrophil 56%, lymphocyte 32%, monocyte 05%, and eosinophils 07%), platelets were 2,24,000/µl and ESR was 110 mm in 1^st^ hour. Serum biochemistry including calcium (10 mg/dl), uric acid (4.0 mg/dl), sodium (141 mg/dl) and potassium (5.0 mg/dl) were normal. Liver function tests showed raised total protein (8.4 gm/dl) and reversed albumin (3.5 gm/dl) and globulins (4.9 gm/dl) ratio. Total bilirubin (0.4 mg/dl), alanine aminotransferase (39 U/L; reference value 30-65 U/L), aspartate aminotransferase (26 U/L; reference value 15-37 U/L) and alkaline phosphatase (51 U/L; reference value 50-136 U/L) were normal. Fasting blood sugar was 107 mg/dl. Renal functions tests showed normal blood urea (12 mg/dl) and creatinine (0.9 mg/dl). Lipid profile was also within normal limits. Patient tested for HIV infection by ELISA was negative.

**Figure 1.**
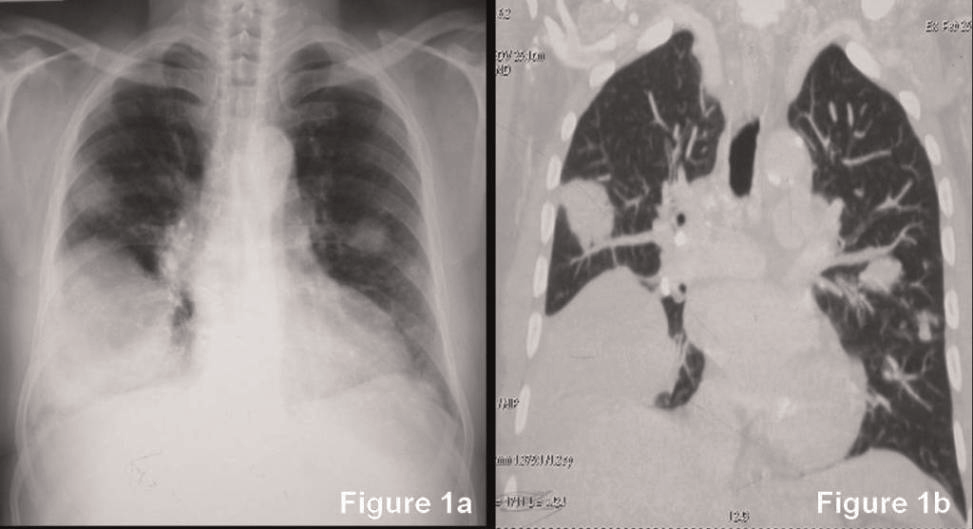
**(a) and (b) Chest radiograph and CT scan (coronal section) of chest.** Chest radiograph **(a)** showing a pleural based mass lesion in right lower zone and nodular lesions in right and left mid zones. Coronal section of CT scan of chest **(b)** showing a large right lower lobe mass and multiple varying size nodules involving left upper lobe, lingula and apical segment of left lower lobe of lung.

**Figure 2.**
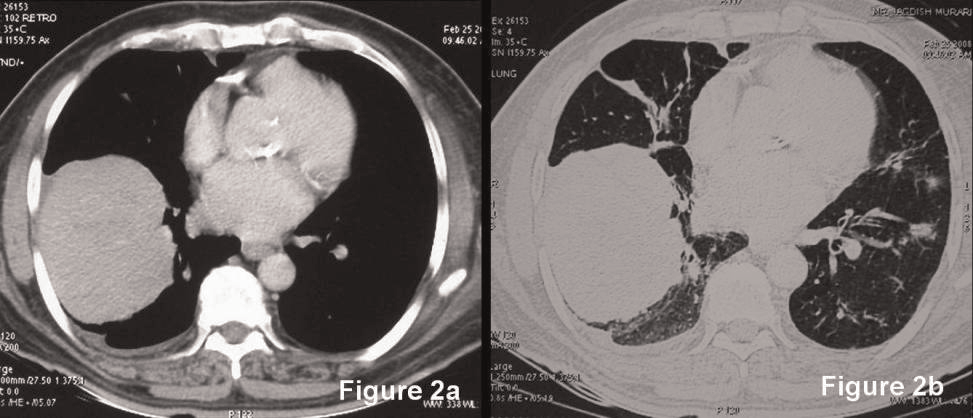
**(a) and (b) Contrast enhanced CT scan of the chest.** Contrast enhanced CT of chest (both mediastinal and lung windows) showing a 9.8 cm × 7.6 cm, homogenous pleural based mass in right lobe of lung and mild right pleural effusion. Multiple varying size nodular lesions involving left upper lobe and apical segment of left lower lobe suggestive of metastatic lung disease.

Chest radiograph (Figure 1a) showed mass lesion in right lower zone and multiple nodules in bilateral lung fields. For further characterization, CT of chest was done. It revealed a large mass with sharp margins in right lower lobe and multiple nodules in both lungs. Multiple nodules of varying sizes were seen in left upper lobe, lingula and apical segment of left lower lobe (Figure 1b, 2a and 2b). It also revealed mild right sided pleural effusion. On the basis of radiological findings, metastatic lung disease from extra thoracic origin, carcinoma lung with metastases were considered as differentials diagnoses. Patient was evaluated for extrathoracic primary site of malignancy. Stool samples for occult blood were negative for three times. Urine did not show any evidence of hematuria. Prostate specific antigen levels were normal. Urine examination was negative for Bence-Jones proteins. Serum electrophoresis revealed M-band and ß-2 microglobulins were raised (1952 µg/l; reference value 510-1470 µg/l) but other tests including bone marrow aspirate and biopsy did not reveal any evidence of plasma cell dyscrasia. Serum LDH was normal.

**Figure 3.**
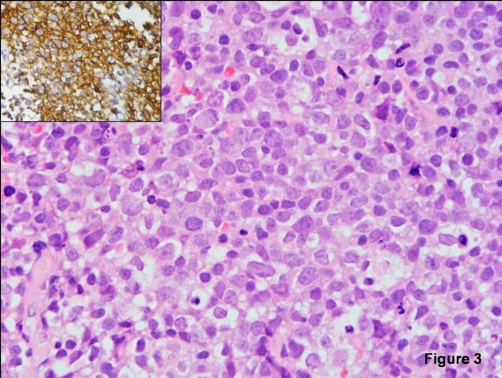
**Photomicrograph showing histopathology of the biopsy specimen.** Photomicrograph of CT guided biopsy from lung mass showing monomorphic round cells with vesicular nucleus and prominent nucleoli (H&E × 400). Inset demonstrates strong diffuse immunostaining for CD20 (LSAB × 200). Findings are suggestive of diffuse large B-cell non-Hodgkins lymphoma.

For the histological diagnosis, CT guided biopsy from the right lung mass was done. Histopathological examination (Figure 3) showed fibrocollagenous tissue infiltrated by monomorphic round cells with scanty cytoplasm, high nucleo-cytoplasmic ratio, large vesicular nucleus and prominent nucleoli in some of the cells. Frequent mitotic activity and apoptotic bodies were noted. Immunohistochemistry showed the cells to be positive for leukocyte common antigen (LCA) and CD20 (B-cell marker) while being negative for CD3 (T-cell marker), cytokeratin and neuroendocrine markers. Stains for acid fast bacilli and fungus were negative. Thus, a final pathological diagnosis of diffuse large cell B-cell lymphoma was rendered. Patient was evaluated for involvement of other sites by lymphoma. Contrast enhanced CT of abdomen did not revealed any mass lesion. Patient was referred to medical oncology for chemotherapy. However, family declined any treatment. Patient last seen a week back was stable.

## Discussion

Primary pulmonary lymphoma (PPL) is defined as a clonal, lymphoid proliferation affecting single or both lungs and that has not spread outside the lungs on diagnosis or in the following 3 months [5]. The term PPL also includes multifocal mucosa-associated lymphoid tissue (MALT) NHL, lung disease with hilar or mediastinal lymph node involvement, and lymphomatoid granulomatosis [1]. World Health Organization classifies PPL into B-cell primary pulmonary NHL, and lymphomatoid granulomatosis [6]. B-cell primary pulmonary NHL are subdivided into low-grade B-cell PPLs (58%-87%), high-grade B-cell PPLs (11%-19%), primary pulmonary plasmacytoma, and intravascular pulmonary lymphomas [1]. The high-grade B-cell PPLs spread rapidly into mediastinal and extra-thoracic locations. This may lead to underestimation of true incidence. Almost 50% of cases coexist with MALT B-cell lymphomas and because of these mixed or transitional forms, it has been hypothesized that high-grade B-cell lymphomas may be the result of the transformation of a low-grade lymphoma. Though cytogenic differences have been found between the 2 types of lymphomas that would seem to rule out this possibility, however, this is still open to debate [1,7]. High-grade B-cell primary pulmonary lymphomas often occur in immunocompromised patients.

Respiratory symptoms, fever, and weight loss are usual presenting symptoms of PPL [1,8]. Usual age of presentation is about 60 years, except in the case of patients with HIV infection who may present earlier [1,8]. The chest x-ray shows a pulmonary mass, nodules, consolidation, atelectasis or pleural effusion. In patients with HIV infection, PPL can present as multiple nodules with cavitation [9]. Fibreoptic bronchoscopy may reveal stenosis due to an exophytic mass or infiltration [8]. For histological diagnosis bronchial or transbronchial or transthoracic fine-needle aspiration or biopsy is required. Histological features include presence of monomorphic atypical lymphoid cells with high mitotic activity, affecting bronchial, vascular, or pleural structures. Necrosis may be evident. Immunohistochemical staining is required to rule out diagnosis of carcinoma, melanoma, or sarcoma. Common immunohistochemical stains used are for lymphoid antigens, epithelial, HMB-45, Melanin-A, actin, S-100 protein and neuroendocrine markers. Lymphomatoid granulomatosis is characterized by angiotropic lesions [1,10].

There is no consensus on treatment. Current treatment options are surgery (for localized lesions), chemotherapy (for bilateral or extrapulmonary involvement, relapse or progression) and radiotherapy (rarely) [11,12]. Single-agent regimens with chloraminophene, cyclophosphamide, azathioprine or steroids have similar efficacy as combination regimens, such as cyclophosphamide, adriamycin, oncovin and prednisone (CHOP) [11,12]. High-grade B-cell lymphomas have shorter survival than for low-grade ones. The average survival period is 8-10 years [11,12] and is shorter for immunocompromised patients.

Our case presented with nonspecific symptom of generalized weakness without any symptom pertaining to respiratory system. As reported previously, PPLs can present with nonspecific symptoms [3,8]. Chest radiograph may be the first clue to the site of disease. Lung mass with multiple nodules in same or other lung is more commonly seen in bronchogenic carcinoma or lung metastases. Sometimes, uncommon malignancies may be encountered which may have better prognosis than the common lung malignancies. In our case, the images were suggestive of metastatic lung disease, but final diagnosis turned out to be a primary pulmonary lymphoma. It further emphasizes the importance of histological diagnosis of lung lesions.

## Conclusion

Primary pulmonary lymphoma is a rare disease and can present with non-specific symptoms. Radiologically, it can easily be confused with commoner malignancies such as, bronchogenic carcinoma with or without metastases. However, it has different therapeutic and prognostic implications. Therefore, physician should put every effort to achieve histopathological diagnosis before prognosticating patient presenting with lung mass.

## List of abbreviations

CT: Computer tomography
ELISA: Enzyme-linked immunosorbent assay
HIV: Human immune deficiency virus
LDH: Lactate dehydrogenase
NHL: Non Hodgkin's lymphoma
PPL: Primary pulmonary lymphoma
